# Early Results of a Novel Hybrid Prosthesis for Treatment of Acute Aortic Dissection Type A With Distal Anastomosis Line Beyond Aortic Arch Zone Zero

**DOI:** 10.3389/fcvm.2022.892516

**Published:** 2022-07-14

**Authors:** Arash Mehdiani, Yukiharu Sugimura, Louise Wollgarten, Moritz Benjamin Immohr, Sebastian Bauer, Hubert Schelzig, Markus Udo Wagenhäuser, Gerald Antoch, Artur Lichtenberg, Payam Akhyari

**Affiliations:** ^1^Department of Cardiac Surgery, Heinrich Heine University Duesseldorf, Düsseldorf, Germany; ^2^Department of Vascular and Endovascular Surgery, Heinrich Heine University Duesseldorf, Düsseldorf, Germany; ^3^Department of Diagnostic and Interventional Radiology, Heinrich Heine University Duesseldorf, Düsseldorf, Germany

**Keywords:** acute aortic dissection type A (AADA), hemiarch and aortic arch replacement, aortic remodeling, *frozen elephant trunk*, AMDS

## Abstract

**Introduction:**

Acute aortic dissection type A (AADA) is associated with high perioperative morbidity and mortality. A novel non-covered hybrid prosthesis (Ascyrus Medical Dissection Stent (AMDS) Hybrid Prosthesis, Cryolife/Jotec, Hechingen, Germany) can be easily implanted to stabilize the true lumen (TL), improve remodeling, and preserve organ perfusion. Although developed for implantation in aortic zone 0, occasionally, partial replacement of the aortic arch and further distal implantation of AMDS may appear favorable. Implantation of AMDS with anastomosis line beyond zone 0 has not been described yet.

**Materials and Methods:**

Between 08/2019 and 12/2020, a total of *n* = 97 patients were treated due to AADA at a single University hospital. Of those, *n* = 28 received an AMDS hybrid prosthesis, of whom in eight patients, due to intraoperative finding the distal anastomosis line was placed distal to the brachiocephalic trunk. Three patients had AMDS implantation in zone I and four were treated by implantation of the prostheses in zone II, and one patient had the implantation performed in zone III. Clinical outcome and the development of a proportional area of TL and false lumen (FL) at defined levels of the thoracic aorta were analyzed.

**Results:**

None of the surviving patients (87.5%) showed signs of clinical malperfusion (i.e., stroke, spinal cord injury, and need for dialysis). A postoperative CT scan showed an open TL in all patients. The proportion of TL with respect to total aortic diameter (TL+FL) was postoperatively significantly higher in zone III (*p* = 0.016) and at the level of T11 (*p* = 0.009). The mean area of TL+FL was comparable between pre- and postoperative CT-scan (*p* = n.s.). One patient with preoperative resuscitation died of multiple organ failure on extracorporeal life support on postoperative day 3.

**Conclusion:**

Implantation of AMDS can be safely performed in patients who need partial replacement of the aortic arch beyond zone 0. The advantages of the AMDS can be combined with those of the total arch repair (remodeling of the arch and prevention of TL collapse) without the possible disadvantages (risk of spinal cord injury).

## Introduction

Acute aortic dissection type A (AADA) remains a life-threatening condition despite the continuous improvement of operative technique and perioperative care for decades (1). Emergency surgery represents the first-line therapy and aims at replacement of the ascending aorta and removal of the primary entry tear (2). An extension of surgery to the aortic arch is preferred when an intimal tear is localized in this region (3–5). In the extreme, total aortic arch replacement (TAR) with concomitant implantation of a stent-graft prosthesis in the descending thoracic aorta (referred to as *frozen elephant trunk*) may be performed, which is currently regarded as the ultimate solution at the technical level to promote favorable aortic remodeling (6, 7). However, real-world data demonstrate a significant increase in 30-day mortality with TAR when compared with the technically more simple replacement for the ascending aorta with an open distal anastomosis, the so-called hemiarch replacement (HAR) (8), while HAR has been associated with an increased risk for aortic re-intervention in the follow-up (9). To simplify the therapy of AADA and make the operation performable for most surgeons irrespective of their specific experience profile, HAR has become a commonly applied technique. Furthermore, according to previous reports, TAR can be performed by nearly all surgeons due to the simplification of the implantation technique, however, an increased risk of stroke remains (10, 11). The Ascyrus Medical Dissection Stent (AMDS Hybrid Prosthesis, Cryolife/Jotec, Hechingen, Germany) has been designed to simplify surgical treatment of the downstream thoracic aorta by implantation line located proximal to the brachiocephalic trunk (zone 0), in addition to the standard replacement of the ascending aorta. Initial short-term results from a multicenter study suggest that the use of AMDS may have a positive impact by (1) sealing the distal anastomotic line to avoid distal anastomotic line new entry (DANE), (2) further supporting positive aortic remodeling by avoiding true lumen (TL) collapse, and (3) also reducing malperfusion associated with AADA (12). However, DANE is only one of several factors contributing to adverse remodeling of the aortic arch and the downstream thoracic aorta. The presence of intimal tear in the aortic arch and a retrograde perfusion of the supra-aortic vessels due to a re-entry further distally in the supra-aortic vessels have been reported as relevant factors requiring an extension of aortic replacement beyond zone 0 (13). Until now, there are reports on the outcome and prognostic value of AMDS implantation distal to the brachiocephalic trunk, i.e., beyond zone 0. It may be hypothesized that such an approach preserves the beneficial impact of AMDS on remodeling of the distal thoracic aorta while reducing the impact of retrograde supra-aortic perfusion of false lumen (FL) and also reducing the proportion of dissected aortic wall in the arch region. This report summarizes our experience with a consecutive series of patients undergoing emergency aortic surgery for AADA involving AMDS implantation in the aortic arch zone I–III.

## Patients and Methods

### Patient's Population and Data Collection

In this study, 97 consecutive patients undergoing cardiac surgery due to AADA at a single university hospital between 08/2019 and 12/2020 were reviewed. Of these, 28 patients (28.9%) received an AMDS hybrid prosthesis at the decision of the attending surgeon. This report analyses the in-hospital course and outcome of those patients in whom AMDS was implanted with the anastomosis line beyond zone 0: three in zone I (between the brachiocephalic trunk and the left common carotid artery, LCCA), four in zone II [between LCCA and left subclavian artery (LSA)], and one in zone III (distal to the LSA) (Table 1).

**Table 1:** Reasons for deviation from zone 0.

**Reason for deviation**	***n* (8)**
Exclusion of intimal tear (re-entry)	7
Dissection of the supra-aortic vessel	7
Ascending proximal aneurysm extending to the arch	2
Circumferential tear of supra-aortic vessel	2
True lumen collapse of the thoracic aorta	3

*There are multiple reasons for deviation. The zone for implantation is chosen by the most distal problem (dissection of supra-aortic vessel or intimal tear in the arch)*.

Demographic data, comorbidities, intraoperative procedures, and postoperative variables of outcome were collected systematically and entered prospectively into a standardized institutional database. Expected mortality was determined using prognostic models from the literature [EuroScore II, Penn, and German Registry for Acute Aortic Dissection Type A (GERAADA) models (14)]. These data were analyzed retrospectively in this study.

## Ascyrus Medical Dissection Stent

The AMDS consists of an uncovered stent part and a proximal felt neck. Both elements intend to readapt the intima against the media and the adventitia and thereby reduce the force of separation between these layers, especially in the area of the anastomosis. This reduces the incidence of DANE and leads to remodeling of the aorta. Due to the fact that the stent is not covered, an occlusion of vertebral vessels is impossible.

### CT Scan

Operative diagnosis was confirmed in all patients based on preoperative contrast-enhanced CT scans covering at least the thoracic cavity. Measurement of the aortic diameter was performed as described before (15) and suggested by STORAGE guidelines (16). Regardless of the localization of intimal tear (entry), the total diameter of the aorta including the aortic wall (such as TL and FL) at the level of zone I and at the level of pulmonary bifurcation was documented. All patients received at least one postoperative CT scan. In all in-hospital survivors, pre-discharge CT scans were performed.

Arch growth was determined in centerline measurement on the planes perpendicular to the aorta using multiplanar CT reformation at the level of zone III. The diameter and the area of TL and FL were measured at the level zone III and at the level of the 11th thoracic vertebrae (T11). For comparison, CT scan images obtained prior to the operation and prior to discharge were analyzed. For a standardized comparison, the ratio of TL area to FL area was calculated and analyzed.

### Surgical Procedure

**Figure 1:**
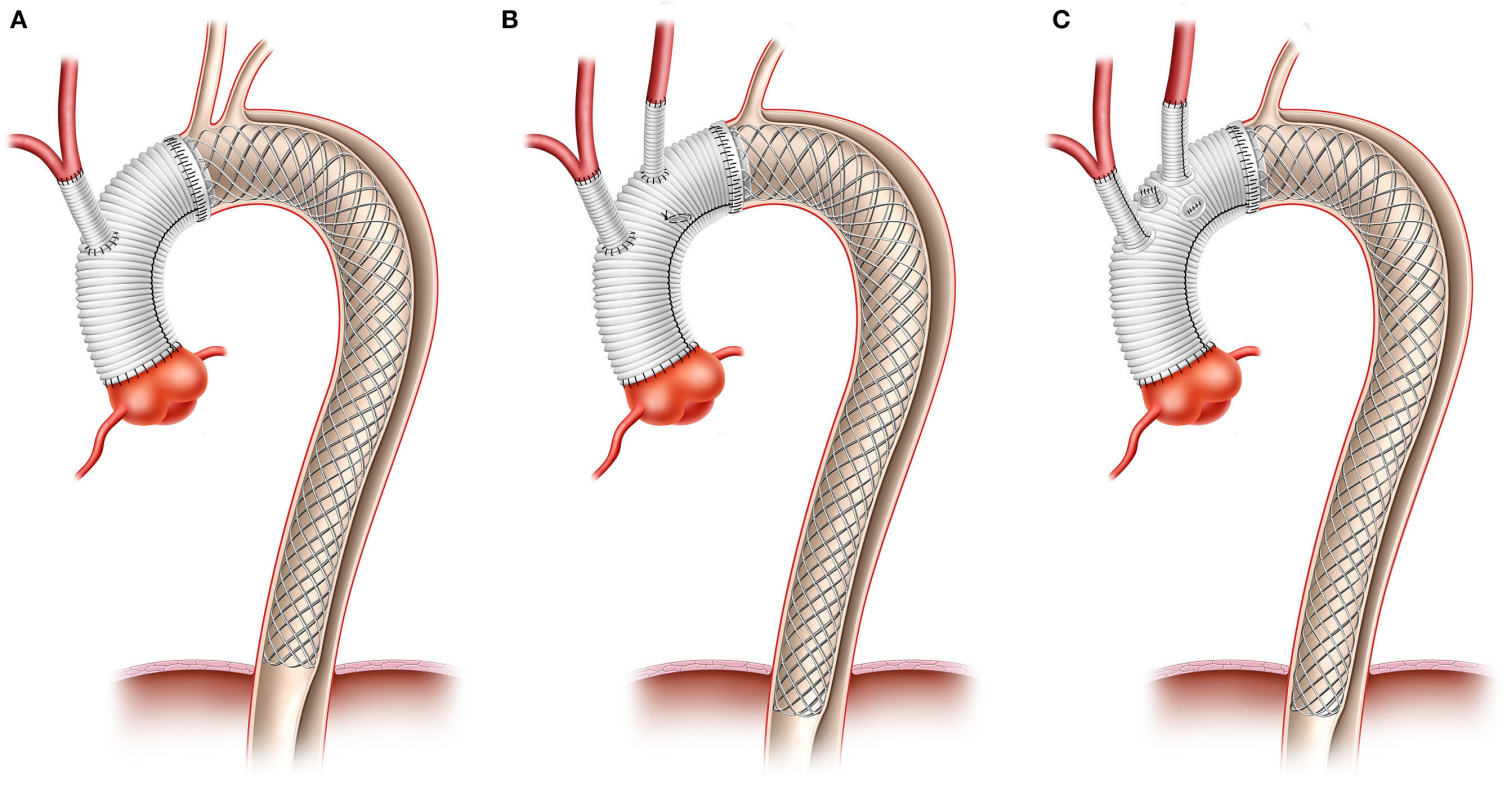
Morphology of different surgical procedure. Anastomosis was ether performed in zone I using a single tube graft for INA **(A)**, or more distally using single tubes for the anastomosis of supra aortic vessels **(B)** or a branched prosthesis **(C)**.

All patients underwent emergency surgery with cardiopulmonary bypass (CPB) initiated with arterial cannulation *via* the right axillary artery and venous cannulation of the right atrium or the right femoral vein. By clamping the innominate artery, lower body circulatory arrest was initiated at a body core temperature of 26–28°C measured in the bladder, while unilateral antegrade cerebral perfusion was achieved with continued perfusion of the right axillary artery. After transecting the ascending aorta, a balloon-inflatable perfusion catheter was endoluminally introduced, and perfusion of the LCCA was achieved for bilateral antegrade cerebral perfusion as the standard technique. Intraoperative near-infrared spectroscopy (NIRS) was used to monitor cerebral oxygenation, particularly during CPB time and hypothermic lower body circulatory arrest (HCA). Further aortic transection was performed up to the intended anastomosis line. AMDS was then implanted using a Teflon stripe on the adventitial aortic side to re-enforce the anastomosis line. Either a branched prosthesis (*n* = 3) or a tube graft (*n* = 5) was used for partial arch replacement. During reperfusion time, supra-aortic vessels were anastomosed to side branches where a branched prosthesis was used, or a small-diameter single tube graft (10 or 12 mm) was employed as an interposition graft for anastomosis of the supra-aortic vessels (Figure 1).

### Ethics Committee Approval

This study was approved by the Ethics Committee of the Medical Faculty of the Heinrich Heine University Düsseldorf (Ref. 2016116135).

### Statistical Analysis

Statistical analyses were performed with the Statistical Package for Social Sciences® (SPSS) 25.0 (IBM, Chicago, USA). Using this program, descriptive statistics and statistical comparisons between CT measurements were performed using the Wilcoxon matched-pairs signed rank test. A *p*-value of < 0.05 was considered statistically significant. Data are presented as mean ± standard error of mean (SEM).

## Results

### Baseline Characteristics

Baseline characteristics are presented in Table 2. While the mean age was 63 ± 15 years, three patients were women (37.5%). No patient had previous cardiac surgery. Furthermore, two patients (25%) presented with prior intubation, of whom one patient was intubated in the setting of cardio-pulmonary resuscitation, while another patient was intubated due to neurological deterioration. Another patient presented with isolated left leg paresis. In addition, two patients were admitted to the hospital due to angina symptoms and ST elevation. The mean predicted 30-day mortality by the GERAADA score was 35.16 ± 18.33%. In the preoperative CT scan, two patients showed pericardial effusion. Angiographic signs of malperfusion were found in all patients.

**Table 2:** Baseline characteristics.

**Characteristics**	**Total number of patients**
**Mean (SD), *N* (%)**	***N*= 8**
Age (years)	63.63 ± 14.98
Gender (female)	3 (37.5)
BMI (kg/m^2^)	29.45 ± 5.23
BSA (m^2^)	2.0 ± 0.3
Hypertension	5 (52.5)
Diabetes	0 (0)
Smoke	4 (50)
Chronic obstructive pulmonary disease	1 (12.5)
Chronic kidney disease	1 (12.5)
Coronary artery disease	2 (25)
Previous aortic pathology	0 (0)
Intubation	2 (25)
Acute shock	1 (12.5)
Preoperative CPR	1 (12.5)
Need of catecholamine	1 (12.5)
**Malperfusion**	
- CT morphologically	8 (100)
**Symptomatically/clinical**	
-Coronary	2
- Neurological	2
- Mesenteric/renal	0
- Extremity	1
**PENN Classification**	
PENN class Aa	2
PENN class Ab	2
PENN class Ac	2
PENN class Ab&c	1
Acute neurological deficit	2 (25)
GERAADA score (%)	35.16 ± 18.33
EuroScore (%)	35.74 ± 19.31
Aortic regurgitation ≥ I°	4 (50)
Left ventricular ejection fraction (LVEF) (%)	44 ± 12
Pericardial effusion	2 (25)

*Baseline characteristics, GERAADA, EuroScore, and the PENN Classification are presented. The PENN Classification refers to a previously described classification of ischemic presentation (17)*.

### Operative Data

Surgical and perioperative data are presented in Table 3. The mean CPB time was 285.25 ± 61.67 min. The lowest body core temperature was 26.2 ± 1.2°C. While lower body HCA time was 65.0 ± 12.6 min and cerebral perfusion time was 158.8 ± 63.2 min. Myocardial protection was achieved with the crystalloid cardioplegic solution in one patient, whereas a cold blood cardioplegic solution was used in the remaining seven patients.

**Table 3:** Surgical and perioperative data.

**Characteristics**	**Total number of patients**
**Mean (SD), *N*(%)**	***N* = 8**
Total operation time	435.9 ± 140.2
CPB time	285.3 ± 61.7
X-clamp time	169.4 ± 60.3
Lower body HCA time	65.0 ± 12.6
Selective antegrade cerebral perfusion time	158.8 ± 63.2
Lowest body core temperature	26.2 ± 1.18
**Cardioplegic solution**	
- cold blood	7 (87.5)
- crystalloid	1 (12.5)
**Root surgery**	
- Aortic valve reimplantation (David)	3 (37.5)
- Root repair	5 (62.5)
**Level of AMDS implantation**	
- Zone I	3 (37.5)
- Zone II	4 (50)
- Zone III	1 (12.5)
**AMDS prosthesis size**	
−40 tubular	1 (12.5)
−40–30 tapered	0 (0)
−55 tubular	3 (37.5)
−55–40 tapered	4 (50)

*CPB, cardio-pulmonary bypass; X-clamp, cross-clamp; HCA, hypothermic circulatory arrest*.

Implantation of the AMDS was performed at the level of aortic arch zone III (*n* = 1), zone II (*n* = 4), or zone I (*n* = 3).

### Postoperative Outcome

Measures of the postoperative outcome are summarized in Table 4. One patient with preoperative shock and cardiopulmonary resuscitation (CPR) needed postoperative extracorporeal life support (ECLS) with a consecutive bleeding disorder and the need for re-thoracotomy. This patient experienced therapy-refractory multiple organ failure and died on postoperative day 3. In-hospital and 30-day mortality were 12.5%. The mean duration of ICU stay was 4.72 ± 2.55 days, patients were discharged from the hospital after an average of 14.86 days. One patient underwent re-intubation due to CO_2_-retention and showed prolonged weaning from ventilator, underwent tracheotomy, and was transferred to respiratory weaning center on postoperative day 13. The mean ventilation time was 2.24 ± 0.88 days. Among surviving patients (7/8), no patient showed relevant postoperative neurological deficit (e.g., stroke and spinal cord injury). All survivors had a Glasgow Coma Scale (GCS) of 15 and a Richmond Agitation Sedation Scale (RASS) of 0 at the time point of discharge.

**Table 4:** Postoperative outcome.

**Characteristics**	**Total number of patients**
**Mean (SD), *N* (%)**	***N* = 8**
Ventilation time (*n* = 7) (h; days)	53.9 ± 21.1 (2.2 ± 0.9)
Duration in ICU (h; days)	113.3 ± 61.3 (4.7 ± 2.6)
Duration on IMC (h; days)	84.3 ± 80.5 (3.5 ± 3.4)
Total hospital stay (days)	14.9 ± 6.8
Need of mechanical circulatory support (central ECLS)	1 (12.5)
Major bleeding	1 (12.5)
Tracheotomy	1 (12.5)
Acute kidney injury with need for hemodialysis	3 (37.5)
Stroke	0 (0)
Spinal cord injury	0 (0)
Postoperative clinical malperfusion	0 (0)
Postoperative still existing dissection of supra-aortic branches	3 (37.5) (patients 3 + 5 + 8)
Perfusion of the false lumen in the arch	2 (25) (patients 5 + 8)
Postoperative true lumen collapse	0 (0)
Postoperative infection	0 (0)
In-hospital death	1 (12.5)
30-day mortality	1 (12.5)

*ECLS, extracorporeal-life support*.

Postoperative CT scans demonstrated that no patient suffered from TL collapse. At the level of the aortic arch, perfusion of the FL was detected in only two patients (arch remodeling). No patients experienced postoperative clinical malperfusion whereby in three patients, a dissection of supra-aortic vessels was detected even after the operation.

### Comparison of CT-Measurements

**Figure 2:**
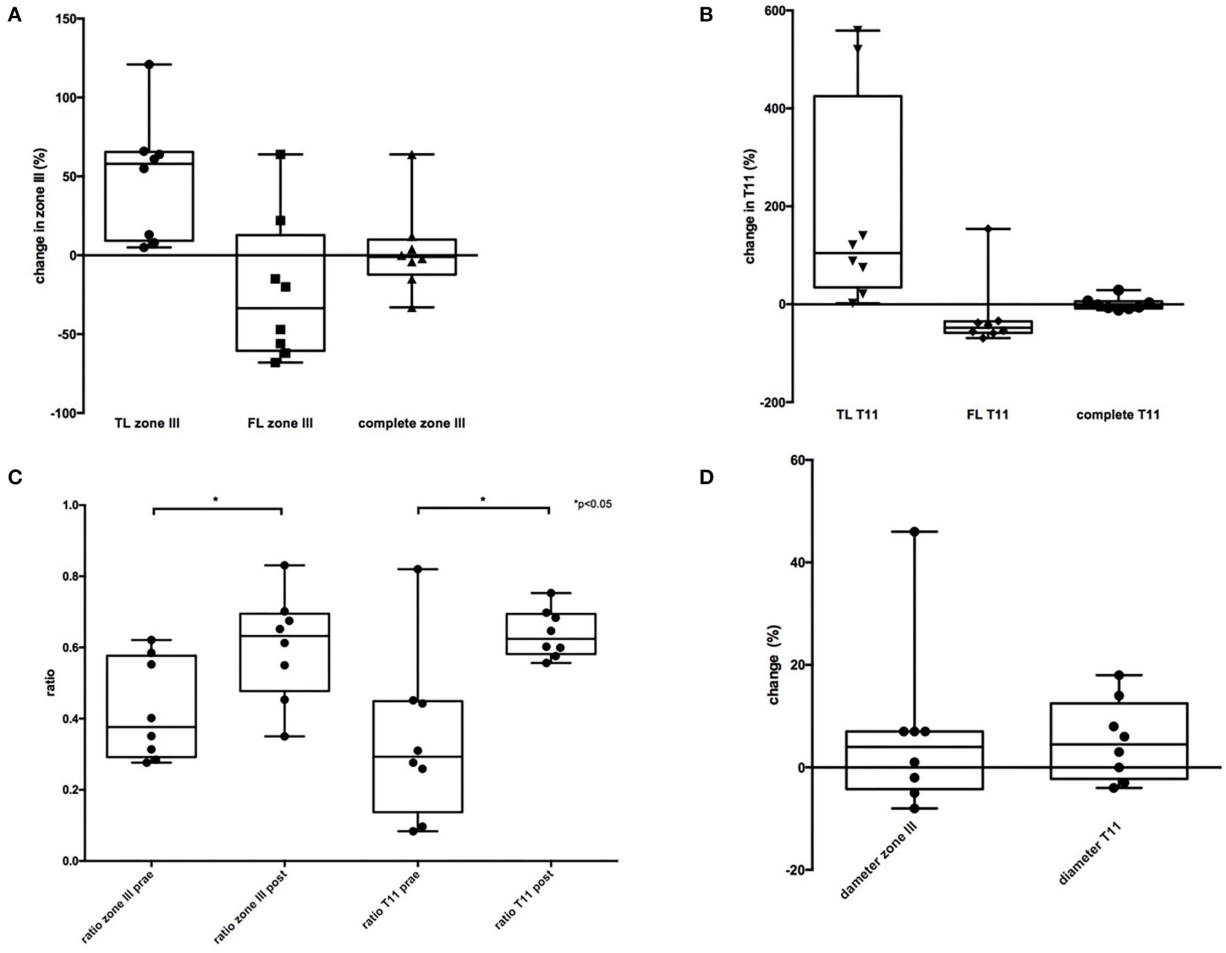
Comparison of CT-measurement. **(A)** Showing an enlargement of the true lumen (TL) and a decrease of the false lumen (FL) in zone III. Area of TL + FL did not changed. **(B)** TL showed a significantly bigger increase in T11 and a significant decrease of the FL. Area of TL + FL did not changed. **(C)** ratio of TL/TL + FL was postoperatively significant higher in both zones compared to preoperative CT. **(D)** total diameter showed no significant difference.

Table 5 shows the CT measurements. Area analyses demonstrated a significant enlargement of the TL in zone III and further distally (T11) when assessing postoperative images as compared with the preoperative status. A decrease in mean cross-sectional area of FL in the latter aortic segments was observed. The ratio between cross-sectional areas of TL and FL, respectively, was significantly higher postoperatively with more than doubled values at the level of zone III. The total diameter increased slightly at both analyzed levels of the thoracic aorta (Figure 2).

**Table 5:** CT measurements.

	**Pre**	**Post**	***p***	**Median change (%)**
Area of TL at zone III (mm^2^)	412.61 ± 201.99	558.12 ± 1,419.60	**0.0078**	**58.32**
Area of FL at zone III (mm^2^)	528.04 ± 104.18	402.38 ± 243.73	0.1953	−34.03
Area of TL + FL at zone III (mm^2^)	940.65+193.75	960.50+276.26	0.9453	−1.81
TL/TL + FL ratio at zone III	0.42+0.14	0.60+0.15	**0.0313**	
Average diameter at zone III (mm)	34.90 ± 4.53	37.10 ± 6.10	0.5156	4.57
Area of TL at T11 (mm^2^)	240.83 ± 150.67	463.81 ± 88.99	**0.0078**	**105.29**
Area of FL at T11 (mm^2^)	484.10 ± 196.58	262.36 ± 69.14	**0.0234**	–**48.46**
Area of TL + FL at zone III (mm^2^)	724.93+101.52	726.17+125.81	0.7422	−3.89
TL/TL + FL ratio at T11	0.34+0.24	0.64+0.07	**0.0234**	
Average diameter at T11 (mm)	31.24 ± 3.06	32.91 ± 3.87	0.1094	4.97

*TL, true-lumen; FL, false-lumen. Bold indicates statistical significant differences*.

## Discussion

In this study, we demonstrate that implantation of an AMDS hybrid prosthesis beyond aortic arch zone 0 is feasible, reproducible, and safe. No patient suffered from postoperative spinal cord injury or malperfusion syndrome. A significant increase in perfused TL could be observed at different segments of the aorta, while a significant reduction of FL cross-sectional area was observed.

Obviously, a shorter operation time can be life-rescuing in the particular emergency settings of surgery for AADA (18). The primary aim of surgery in those patients with significantly impaired outcomes is early survival. Furthermore, the freedom of re-intervention should be aimed for as surgery and interventional procedures are associated with further risk of mortality and morbidity.

Hemiarch repair is associated with a shorter CPB time and operation time. However, the need for a second operation can be possible (9, 19–21). While total arch replacement with *the frozen elephant trunk* allows for the distal extension of the stent and allows sealing of potential distal intima tears. At the same time, covered stents can lead to spinal artery occlusion and spinal cord injury (22). In addition, neurological complications, e.g., stroke and TIA, can be higher in patients treated with TAR (7, 23). However, few AADA centers describe the feasibility of TAR due to a simplified operation technique with a proximalized distal anastomose line, in the acute setting of aortic dissection. Still, many centers prefer HAR over more complex surgery involving extended aortic arch replacement with or without stent graft implantation (TAR).

The AMDS hybrid prosthesis consists of an uncovered stent part and a proximal felt neck located at the level of the anastomotic line. Therefore, this device represents a hybrid solution intended for implantation at aortic zone 0 with technical complexity compared to that of the hemiarch replacement. However, the first clinical results suggest that an additional benefit of aortic remodeling with reduced malperfusion and a lower rate of DANE speak in favor of the implementation of AMDS (12, 24).

A few scenarios can make the use of the AMDS in the first moment impossible (Table 1). However, by shifting the distal anastomosis line more distally, AMDS could also be implanted in cases with focal re-entry in the aortic arch region. By careful preoperative assessment of the CT scan, preoperative determination of surgical strategy and location of the anastomotic line may be possible in the majority of patients presenting with AADA. The results presented herein show that this technique is a safe and reproducible alternative to TAR, combining the benefits from simple hemiarch with some benefits otherwise observed with the more complex total arch replacement. Intimal tears in the arch region could be resected, dissected supra-aortic vessels could be repaired in most patients, TL collapse could be prevented in all patients, and the risk of spinal cord injury was virtually omitted. We could also confirm the results of Bozso et al. (24), that by using the ADMS, DANE could be prevented and no antegrade perfusion was performed, which also led to the remodeling of the aorta.

The CT analysis showed a significant increase in the TL at the level of zone III and T11 in our cohort. No TL collapse was observed in any part of the aorta. The area of FL was reduced in six patients in zone III and seven patients in T11, leading to a relatively stable total area in both zones, indicating remodeling of the arch and the descending aorta. The ratios of TL/TL+FL were significantly higher in both zones, which may be regarded as evidence of favorable remodeling. While the ratio in preoperative CT scans is below 0.5 (meaning the proportion of TL compared with the entire aortic cross-sectional area is <50%), the TL proportion was increased in postoperative CT scans (ratio > 0.5).

In two patients, an increased aortic diameter on postoperative CT with growth of FL area at the level of aortic zone III and T11 was observed. In these patients, a persistent dissection of supra-aortic vessels (in particular of the LSA) was noted. We speculate that a suspected entry/re-entry in that vessel may cause a retrograde flow into the FL and thus may prevent a positive remodeling of the downstream thoracic aorta.

Preoperative CT scans are often not sensitive enough for identifying additional intima tears in the supra-aortic vessels. Recognized dissected supra-aortic vessels should be anastomosed separately to further enforce favorable arch remodeling. However, the associated increase in technical complexity may have a negative impact on the overall outcome, which will have to be addressed in future studies.

The purpose of this study was not to compare TAR with AMDS due to different patient selection criteria. The aim of the study was rather to show that in patients where a standard implantation of AMDS at the level of aortic zone 0 is not favored, a movement of the implantation site distally may be a solution to preserve the advantages associated with the implantation of the hybrid stent. For the first time, we could show that intimal tears in the arch region or circular re-entry sites at supra-aortic vessels can be treated using this strategy. However, a meticulous study of the preoperative CT scan is of paramount value for accurate strategy planning as well as to identify possible reentries in supra-aortic vessels, which may cause postoperative growth of FL and may lead to further need for intervention.

## Limitation

This study has several limitations. First, this is a retrospective observational study with a small number of patients. In addition, only immediate results are demonstrated; follow-up and long-term results are needed to evaluate the role of the presented approach for sustainable positive arch remodeling, especially in those two patients with the growth of the FL in the early period. As a standard, follow-up visits at 3, 6, and 12 months were presumed but could not be realized in all patients due to incompliance. Furthermore, longitudinal observation of a larger cohort is necessary.

## Conclusion

The implantation of the novel hybrid prosthesis can be performed safely and reproducibly at the aortic arch levels beyond zone 0 in patients with AADA. Applying this approach, an early remodeling of the aortic arch and the prevention of TL collapse with no risk of spinal cord injury can be achieved.

## Data Availability Statement

The raw data supporting the conclusions of this article will be made available by the authors, without undue reservation.

## Ethics Statement

The studies involving human participants were reviewed and approved by Ethics Committee of the Medical Faculty of the Heinrich Heine University Düsseldorf (Ref. 2016116135). Written informed consent for participation was not required for this study in accordance with the national legislation and the institutional requirements.

## Author Contributions

AM and PA conceived the concept of the present study and drafted the paper. LW, SB, and YS contributed with data collection. MI and GA supported CT-data acquisition and data processing. MW, AL, and HS contributed to study design. All authors took part in both data interpretation and critical revision of the manuscript.

## Funding

This study was supported by institutional grants from the Department of Cardiac Surgery, University Hospital and the Medical Faculty of Heinrich Heine University Dusseldorf.

## Conflict of Interest

PA and MW have received speaker fees from Cryolife. The remaining authors declare that the research was conducted in the absence of any commercial or financial relationships that could be construed as a potential conflict of interest.

## Publisher's Note

All claims expressed in this article are solely those of the authors and do not necessarily represent those of their affiliated organizations, or those of the publisher, the editors and the reviewers. Any product that may be evaluated in this article, or claim that may be made by its manufacturer, is not guaranteed or endorsed by the publisher.
